# Effect of crocin, an active saffron constituent, on ethanol toxicity in the rat: histopathological and biochemical studies

**DOI:** 10.22038/IJBMS.2019.37133.8845

**Published:** 2020-01

**Authors:** Alireza Rezaee-Khorasany, Bibi Marjan Razavi, Elahe Taghiabadi, Abbas Tabatabaei Yazdi, Hossein Hosseinzadeh

**Affiliations:** 1 Pharmaceutical Research Center, Pharmaceutical Technology Institute, Mashhad University of Medical Sciences, Mashhad, Iran; 2 Targeted Drug Delivery Research Center, Pharmaceutical Technology Institute, Mashhad University of Medical Sciences, Mashhad, Iran; 3 Department of Pharmacodynamics and Toxicology, School of Pharmacy, Mashhad University of Medical Sciences, Mashhad, Iran; 4 Orthopedic Research Center, Shahid Kamyab Hospital, Mashhad University of Medical Sciences, Mashhad, Iran; 5 Ghaem Hospital, Department of Pathology, Mashhad University of Medical Sciences, Mashhad, Iran

**Keywords:** Crocin, Ethanol, Hepatotoxicity, Nephrotoxicity, Oxidative stress

## Abstract

**Objective(s):**

The drinking of ethanol causes the wide range of clinical illness and morphological changes including hepatotoxicity and nephrotoxicity. In the current study, the protective properties of crocin versus oxidative stress, apoptosis and inflammation induced by ethanol were assessed.

**Materials and Methods:**

The male Wistar rats were divided into seven groups consisting of 6 rats in control, ethanol (50% v/v - 5 g/kg), crocin (10, 20 and 40 mg/Kg) plus ethanol, crocin 20 and 40 mg/Kg.

**Results:**

The MDA level was remarkably enhanced, while the content of GSH was significantly diminished in the kidney and liver of alcoholic rat but protective groups restored the level of MDA and GSH contents. Ethanol consumption induced hepatotoxicity and nephrotoxicity as evidenced by biochemical abnormalities and histopathological damages but crocin improved them. Also, crocin restored the TNF-α and IL-6 levels in the liver. The consumption of ethanol enhanced the levels of caspase-3, -8, -9 and Bax/Bcl-2 ratio (mRNA and protein) but, western blot and real-time PCR data confirmed that crocin treatment prevented apoptosis induced by ethanol.

**Conclusion:**

This research demonstrates that crocin has protective activities against ethanol toxic effects in rat liver and kidney via anti-inflammatory, antioxidant, and anti-apoptotic effects.

## Introduction

Ethanol is an addictive agent that can derive from the fermentation of crops such as grains, vegetables and fruits (1). Ethanol consumption leads to various toxic and pharmacological impacts on numerous organs such as brain, liver, heart and kidney (2). Also, ethanol-related different sicknesses such as testicular injury, myopathy, cerebellar atrophy, cancer, immune deficiency, physical dependence and neuropsychiatric problems have been reported (3). Metabolism of ethanol in liver is substantially affected by three enzymes: microsomal ethanol oxidizing system (MEOS), catalase (CAT), and ethanol dehydrogenase (ADH) (4). It has been acknowledged that metabolism of ethanol in liver can cause the appearance of reactive oxygen species (ROS). During this process, extremely free radicals are generated which are proved to be detrimental. Hence, liver is considered as the initial organ, which is susceptible to harm (5). Due to destructive effects of ethanol, some abnormalities are observed in fat and carbohydrate metabolism, hepatic lipoprotein release and mobilization of fat stores, hepatic absorption of circulating lipids, Krebs cycle function and oxidation of fatty acid (6). All these alterations lead to triglycerides accumulation in hepatocytes, steatosis, and fibrosis, as well as high level of serum triglycerides (7). Kidney is another target organ that its function is disturbed by ethanol. Ethanol causes several detrimental impacts on kidney such as necrosis, decrement of glomerular filtration rate, abnormal levels of protein and glucose in urine, and concentrations of creatinine and blood urea (8). According to studies, an imbalance in free radical formation, antioxidant defense systems, and production of ROS is induced by ethanol metabolism, which results in oxidative stress which is regarded as a substantial harm in cell (9). 

Thus, it is verified that ethanol, affected by oxidative stress, produces a toxin which has a detrimental role in liver and kidney of animals as well as humans. In such conditions, it is impossible to avoid the appearance of lipid peroxidation. Furthermore, the extrinsic and intrinsic signaling pathways stimulate the apoptosis as a result of toxicity impacts of ethanol (10). The aforementioned findings demonstrated that the antioxidant balance level in liver and kidney was disturbed by ethanol or its metabolite.

Growing bodies of research ratified that antioxidants illustrated significant and helpful impacts on declining the occurrence of detrimental alterations caused by ethanol (11). The current interest lies in discovering fresh antioxidants from natural sources rather than artificial antioxidants in medicinal chemicals and food processing. Nowadays, according to different studies saffron is known as a potent and phenomenal natural antioxidant. 

Saffron, dried dark red stigmas, is a member of Crocoideae from Iridaceae family, which is found in the flower of the plant *Crocus sativus,* L. Picrocrocin, safranal, and crocin are the most important pharmacological components in saffron. Saffron color is due to the crocin consists of crocetin and two sugars. Picrocrocin, one of the ingredients of saffron, produces a bitter taste. In addition, aroma and odor of saffron are produced through hydroxylation of picrocrocin into a kind of oil known as safranal (12). According to studies conducted so far, modern pharmacological investigations suggest that crocin has numerous therapeutic impacts on different parts of body namely central nervous system (anti-Alzheimer and anti-Parkinson, antidepressant and anticonvulsant) (13-17), cardiovascular system (18, 19), immune system (20-22), genital system (23) and eye (24, 25). Furthermore, several crocin impacts include chemoprotective aspects, treatment of digestive disorders, anti-cancer, and anti-genotoxic (12). Regarding to these facts, the prophylactic and therapeutic roles of saffron and its constituents have been confirmed and are due to their beneficial properties including antioxidant and anti-apoptotic effects (15). Based on the aforementioned results, the current study intends to investigate the *in vivo* defensive impacts of crocin against apoptosis, inflammation, and oxidative stress induced by ethanol usage in rats’ liver and kidney.

## Materials and Methods

***Chemicals***

Absolute ethanol, sodium dodecyl sulphate (SDS), N, N, N, N-tetramethyl-ethylenediamine (TEMED), thiobarbituric acid (TBA), β-mercaptoethanol (β-ME), malondialdehyde (MDA) were purchased from Merck (Germany). Complete protease inhibitor cocktail, reduced glutathione and phenyl-methanesulfonyl fluoride (PMSF) were supplied from Sigma-Aldrich Chemical Company (USA). BCA protein assay kit (USA, Rockville) and enhanced chemiluminescence Western blotting detecting reagents (ECL) were provided from Pierce. Coomassie (Bradford) Protein Assay Kit (USA, New York) and polyvinylidene difluoride (PVDF) membrane were prepared from Bio-Rad. TNF-α Rat ELISA Kit (USA, Salem), IL-6 Rat ELISA Kit (USA, San Diego), and Express one-step SYBR R Green ER™kit (USA, Carlsbad) were purchased from Invitrogen.

***Preparation of crocin from saffron stigmas***

Saffron stigma was prepared from Novin Saffron Company, Mashhad, Iran. After grinding, saffron stigma powders (10 g) were suspended in 20 ml ethanol 70% at 0 ^°^C. It was shaken by vortex (VTX-3000, Scientfica, Japan) for 3 min. The supernatant was separated after centrifugation (Universal 320R, Heittich, Germany) at 3000 g for 10 min. Ethanol (70% - 20 ml) and sediment were mixed and extraction should be repeated. This step was repeated five other times. A glass container was used for keeping of the solution at -18 ^°^C for one month in darkness. After separation of collected crystals from solution, to remove remaining water, crystals were washed with acetone (26, 27). The efficiency of crocin extraction was 16%.

***Animals***

Forty-two adult male Wistar rats (weight 190-240 g) were prepared by Animal Center of School of Pharmacy, Mashhad University of Medical Sciences. The experimental animals were maintained in plastic cages with a 12 hr light/dark cycle. Standard conditions including free availability of water and food and suitable temperature (23±2 ^°^C) were prepared throughout the four weeks. All animal studies were done in accordance with Mashhad University of Medical Sciences, Ethical Committee Acts (No=910552).

***Experimental design***

For evaluation of ethanol dose and percentage, a pilot study was done. The best result was 50% v/v and 5 g/kg for ethanol. The selected doses of crocin were 10, 20 and 40 mg/kg that was according to previous scientific studies (28, 29). After the stabilization of ethanol dose and percentage and doses of crocin, the experimental animals were divided into seven groups and each group consisted of six rats for the main experiment. For intraperitoneally administration, fresh crocin was dissolved in distilled water. Also, to prepare ethanol 50% v/v, distilled water was used. Distilled water was also used for group 1 (control group) as a control group by gavage. Group 2 received ethanol (50% v/v - 5 g/kg) orally by gavage. Groups 3, 4 and 5 were administered crocin (10, 20 and 40 mg/kg) plus ethanol (50% v/v - 5 g/kg). Groups 6 and 7 were treated with crocin 20 mg/kg and crocin 40 mg/kg, respectively. All groups of animals have been received daily treatment for four weeks. At the end, after overnight fast, the rats were killed by decapitation. After the occurrence of blood clotting, the samples were centrifuged at 4000 g for 15 min and the obtained serums were maintained at -20 ^°^C for further biochemical analysis. For histopathological detection, some parts of liver and kidney were washed in normal saline and preserved in neutral buffered formalin (10%) and other parts of kidney and liver were stored at -80 ^°^C for next analyses. 

***Biochemical blood evaluation***

The commercial colorimetric kits were used for measurement of serum levels of AST, ALT, and ALP as liver enzymes. Also, urea, creatinine, HDL, LDL, TG, FBS, and total cholesterol were evaluated with standard diagnostic kits (Hitachi, Japan).

***Histopathology***

Tissue samples of liver and kidney were fixed in 10% buffered formalin solution for at least 24 hr, passaged of samples by Autotechnicon (overnight) and then embedded in paraffin blocks. Rotatory microtome was used to cut the tissue samples at 5 μm thickness, mounted on a glass slide, stained with hematoxylin and eosin. Then, they were dehydrated, cleared and cover-slipped and ultimately, the slides of samples were examined under the light microscope. Scores between normal histology, mild (+), moderate (++), and severe (+++) were devoted to range of histopathological damage in liver and kidney tissues.

***Assessment of MDA in the kidney and liver tissues***

MDA is the famous indicator of oxidative stress and lipid peroxidation biomarker. The method of Niehaus and Samuelsson was conducted for determination of MDA level (30). For assessment of MDA, it should be prepared a homogenate (10% - w/v). Therefore, tissues were homogenized in cold 1.15% KCl at 4 ^°^C for 2 min. Then, the homogenate (0.5 ml), TBA solution (0.6%, 1 ml) and phosphoric acid (1%, 3 ml) were mixed and bath of boiling water was used to incubate the mixture for 45 min. After that, the mixture was maintained in cold water and the sample temperature decreased to room temperature. N-butanol (4 ml) was added to the mixture and the obtained combination was stirred for 2 min and then, centrifuged for 10 min at 3000 g. The tube of butanol phase (supernatant) was replaced a new tube, and at the end, supernatant absorbance was read by spectrophotometer at 532 nm (Jenway 6105 UV/Visible, Jenway, UK). The content of MDA was expressed as nmol/g tissue.

***Measurement of GSH content in the kidney and liver tissues***

GSH was determined by the method of Moron *et al* in the kidney and liver (31). For preparation of homogenate (10% - w/v), ice-cold 0.1 M phosphate buffer (pH=7.4) was added to the tissues and the mixture was homogenized. GSH contents reduction were measured using 5, 5´-dithiobis (2-nitrobenzoic acid) (DTNB) which produced 5-thio-2-nitrobenzoic acid (TNB) that have the yellow color. The equal amounts of samples and 10% trichloroacetic acid (TCA) were mixed and the resulting combination was centrifuged at 3000 g for 5 min. Then, supernatants (0.5 ml), DTNB reagent (0.04%, 0.5 ml) and sodium phosphate buffer (0.1 M, pH=8.0, 2 ml) were added. Ultimately, UV-VIS spectrophotometer was applied to determine the TNB absorbance at 412 nm. The content of GSH was assessed in accordance with a standard curve and it was expressed as nmol/g tissue.

***Assessment of inflammatory biomarkers***

Invitrogen ELISA kits was used for assessment of the specific inflammatory biomarkers (TNF-α and IL-6) in the liver and kidney tissues according to the instructions of the manufacturer and ELISA reader (Stat fax-2100, UK).

***Western blot analysis***

For Western blot analysis, pieces of liver and kidney were used for extraction of the protein. The samples were homogenized in the homogenization buffer containing 10 mM NaF, Tris 50 mM pH = 7.4, 1 mM Na_3_VO_4_, 2 mM EDTA, 10 mM b-glycerol-phosphate, 1 mM phenylmethylsulfonyl fluoride (PMSF), 0.2% W/V sodium deoxycholate and complete protease inhibitor cocktail (Sigma, P8340) using Polytron homogenizer (POLYTRON- PT 10-35, Kinematica, Switzerland) in ice and the obtained combination was centrifuged at 4 ^°^C and 10000 g for 15 min. The determination of total protein content was performed by using Bradford protein assay kit (Bio-Rad). Also, immune blotting analysis was conducted for assessment of the levels of β-actin, caspase-8, -9, -3, Bax and Bcl2. Equal amounts of protein extracts (50 μg) and loading buffer were mixed and then heated at 95 ^°^C for 5 min and loaded to SDS-PAGE gel. The mixture was separated by sodium dodecyl sulfate-polyacrylamide gel electrophoresis on a 12% gel and transfer of proteins to polyvinylidene fluoride (PVDF) membrane was done in the next step. The blots were incubated in blocking buffer TBS-T (0.1% Tween-20 and 5% nonfat skim milk in Tris-buffered saline) for 2 hr at room temperature. The primary and secondary antibodies were observed in the Table 1. All antibodies were used at a dilution of 1:1000. The incubation time for the primary antibodies at room temperature was 2 hr and then the blots were washed 3 times for 5 min each in TBS with 0.1% Tween-20. The corresponding secondary antibody was used for incubation of the membranes for 60 min. Protein bands were visualized by Alliance gel doc (Alliance 4.7 Gel doc, UK) and Enhanced chemiluminescence (Pierce ECL Western blotting substrate). UV Tec Software (UK) was used to semi-quantify protein bands. All protein bands were normalized against β-actin protein to reach the result.

***Isolation of RNA and real-time PCR (RT-PCR)***

Isolation of total RNAs from kidney and liver tissues was done by high pure RNA tissue kit (Roche, #12033674001) according to the manufacturer protocol. For evaluation of quantity and quality (260/280 and 260/230 ratios) of isolated RNAs, Nanodrop (NanoDrop™ 2000, USA) was used and then, samples of RNAs were kept at -80 ^°^C (Freezer -80, JAHL JD150L, UK). To determination of transcript levels, step one thermal cycler (ABI) and Express one-step SYBR Green ER™kit (Invitrogen, #11780–200) were performed. For evaluation of β-actin (NM_031144.3), Bcl2 (NM_016993.1), and Bax (NM_017059.2), Beacon Design® software (BioSoft) was considered to select the primer pairs (Table 2). Melting curve analysis was prepared for evaluation of products and primers quality. To normalize the relative quantitation values of the genes, β-actin was used as a criterion. Increasing of genes expression was measured by ΔΔCT method.

***Statistical analysis***

The results are demonstrated as mean ± SEM. ANOVA followed by Tukey-Kramer test were used to compare means. *P*-values less than 0.05 were regarded as significant.

## Results

***Effect of ethanol and crocin on body weight changes***

**Figure 1:**
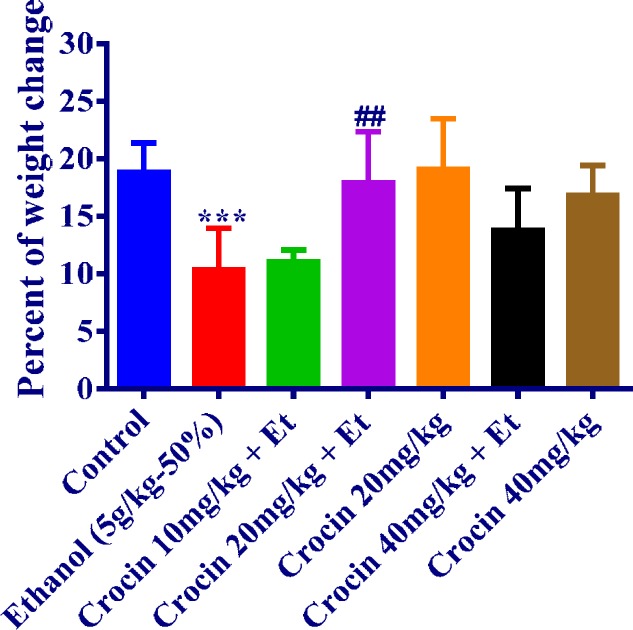
Effects of ethanol and crocin treatment (4 weeks) on body weight changes. Crocin was administered intraperitoneally, once a day. Ethanol (Et) was administered via gavage to rats once a day. Data showed as mean±SEM, *** *P<*0.001comparison with control, ## *P<*0.01 comparison with ethanol-treated group, Tukey-Kramer test, n=6

The body weight alterations and weight gain were evaluated for each week during the study. The lowest weight gain was seen in ethanol group (*P<*0.001). The difference between ethanol group and group of crocin 20 mg/kg plus ethanol was meaningful, while weight gain in group of crocin 20 mg/kg and control group was not considerable (Figure 1).

***Biochemical evaluation***

The levels of AST, ALT, LDL, TG, urea and creatinine were enhanced in the ethanol-treated group in comparison with the control group (*P<*0.05, *P<*0.001). Crocin 20 mg/kg plus ethanol reduced urea, creatinine, AST, ALT, LDL and TG levels which increased by ethanol (*P<*0.05, *P<*0.01, *P<*0.001). The effects of ethanol on cholesterol, FBS, HDL and ALP levels was not significant (Table 3).

***Measurement of MDA level and GSH content in the liver and kidney tissues***

**Figure 2:**
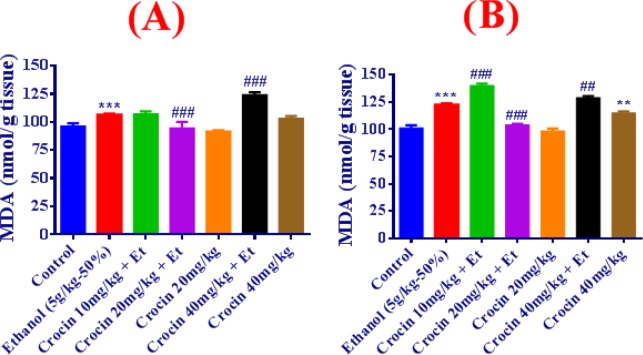
Effects of ethanol (Et) and crocin treatment (4 weeks) on MDA level in the liver (A) and kidney (B) in rats. Crocin was administered intraperitoneally, once a day. Ethanol was administered via gavage to rats once a day. Data showed as mean±SEM, ** *P<*0.01, *** *P<*0.001 comparison with control, ### *P<*0.001 comparison with ethanol-treated group, Tukey-Kramer test, n=6

**Figure 3:**
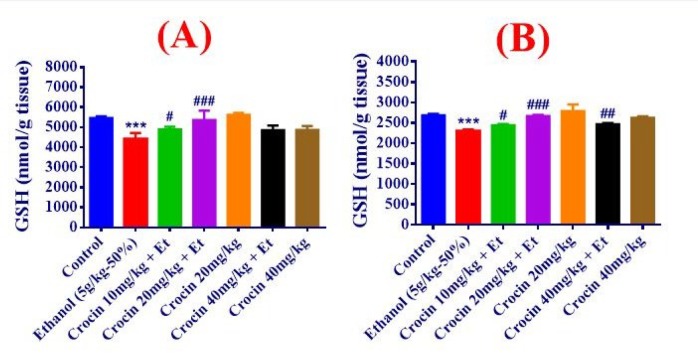
Effects of ethanol (Et) and Crocin treatment (4 weeks) on GSH content in the liver (A) and kidney (B) in rats. Crocin was administered intraperitoneally, once a day. Ethanol was administered via gavage to rats once a day. Data showed as mean±SEM, * *P<*0.05, ** *P<*0.01, *** *P<*0.001 comparison with control, ### *P<*0.001 comparison with ethanol-treated group, Tukey-Kramer test, n=6

The GSH content was considerably reduced by ethanol, while the enhancement of MDA level was significant in comparison with control group in the liver and kidney of ethanol-treated rats (*P<*0.01, *P<*0.001). Simultaneous usage of crocin 20 mg/kg and ethanol could improve the MDA level and GSH contents compared to the control group (Figure 2, Figure 3). 

***Histopathological analysis***

**Figure 4:**
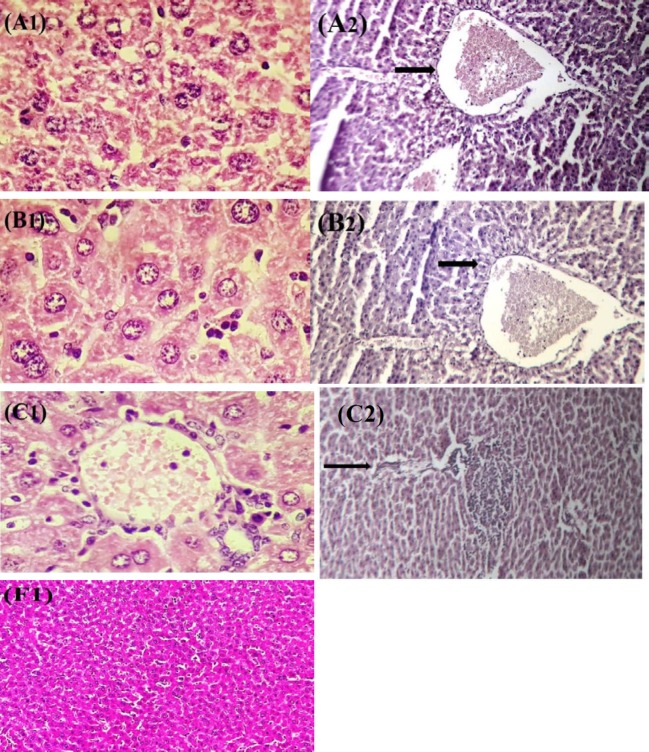
Effect of crocin and ethanol (Et) on histopathological changes in liver tissue of rat after 4 weeks treatment. (A1) Crocin 20 mg/kg+ethanol group (Hematoxylin and eosin, 40×) and (A2) ethanol treated rats showed severe congestion (Hematoxylin and eosin, 40×). (B1) Liver of crocin 20 mg/kg+ethanol group (Hematoxylin and eosin, 100×) and (B2) ethanol treated rats showed steatosis (Hematoxylin and eosin, 40×). (C1) Liver of crocin 20 mg/kg+ethanol group (Hematoxylin and eosin, 100×) and (C2) ethanol treated rats showed portal lymphocytic infiltration (Hematoxylin and eosin, 40×). (F1) Normal liver (Hematoxylin and eosin, 40×)

**Figure 5:**
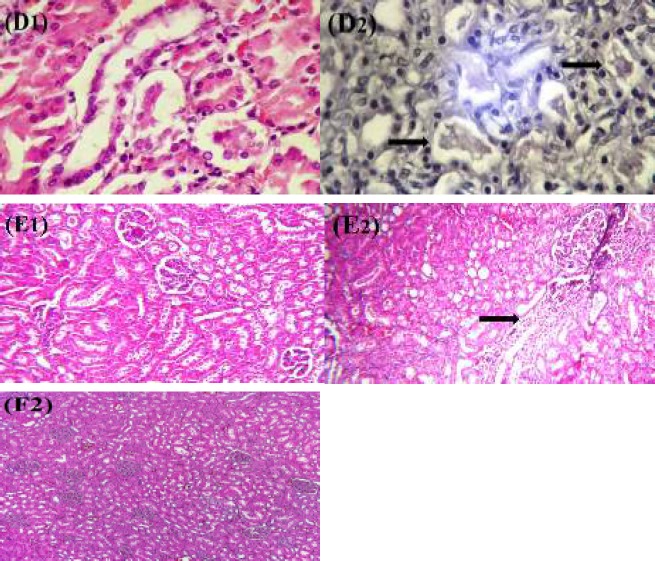
Effect of crocin and ethanol (Et) on histopathological changes in kidney tissue of rat after 4 weeks treatment. (D1) Crocin 20 mg/kg+ethanol group (Hematoxylin and eosin, 100×) and (D2) ethanol treated rats showed proteinuria (Hematoxylin and eosin, 100×). (E1) Kidney of crocin 20 mg/kg+ethanol group (Hematoxylin and eosin, 100×) and (E2) ethanol treated rats showed infiltration of inflammatory factors around glomerular (Hematoxylin and eosin, 100×). (F2) Normal kidney (Hematoxylin and eosin, 40×)

The control group and crocin 20 mg/kg alone had the normal histological findings in the kidney and liver (Table 4, Figure 4, Figure 5). Histological damages induced by ethanol were seen in the kidney and liver tissues. Ethanol-induced liver damages were severe congestion (dilatation in the central vein of the lobule), steatosis and portal lymphocytic infiltration (Table 4 and Figure 4-A2, Figure 4-B2, Figure 4-C2). The histopathological damages of kidney such as proteinuria and infiltration of inflammatory factors around glomerular were observed in ethanol group (Table 4 and Figure 5-D2, Figure 5-E2). In contrast to the ethanol group, the kidney and liver lesions were improved in group of crocin 20 mg/kg plus ethanol (Table 4). In other words, histopathological changes in groups of crocin 20 mg/kg plus ethanol and control were the same (Table 4 and Figure 4-A1, Figure 4-B1, Figure 4-C1, Figure 5-D1, Figure 5-E1).

***Inflammatory markers measurement***

**Figure 6:**
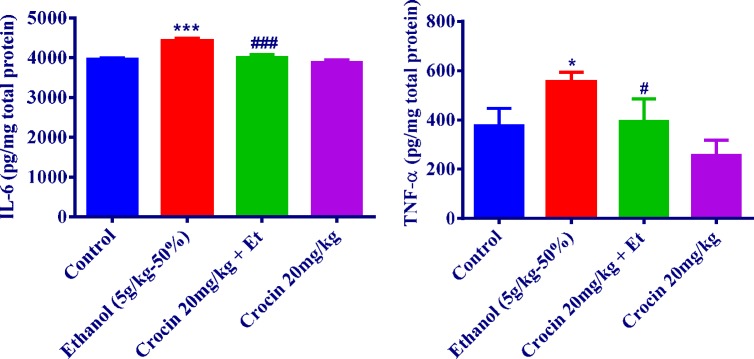
Effects of ethanol (Et) and crocin treatment (4 weeks) on IL-6 and TNF-α levels in the rat liver. Crocin was administered intraperitoneally, once a day. Ethanol was administered via gavage to rats once a day. Data showed as mean±SEM, * *P<*0.05, *** *P<*0.001 comparison with control, # *P<*0.05, ### *P<*0.001comparison with ethanol-treated group, Tukey-Kramer test, n=6

**Figure 7:**
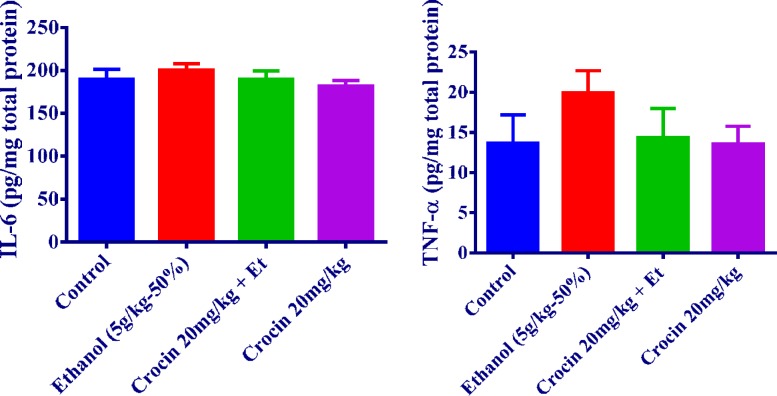
Effects of ethanol (Et) and crocin treatment (4 weeks) on IL-6 and TNF-α levels in the rat kidney. Crocin was administered intraperitoneally, once a day. Ethanol was administered via gavage to rats once a day. Data showed as mean±SEM, Tukey-Kramer test, n=6

As seen in Figure 6, the production of IL-6 and TNF-α in ethanol treatment group had an increasing trend as compared with the control group in the liver (*P<*0.001 and *P<*0.05, respectively). Crocin 20 mg/kg plus ethanol decreased the concentration of TNF-α and IL-6 to those of the control group (Figure 6). It is noteworthy that there were no remarkable changes in TNF-α and IL-6 levels in the rat kidney (Figure 7).

***Western blot analysis for apoptosis***

**Figure 8:**
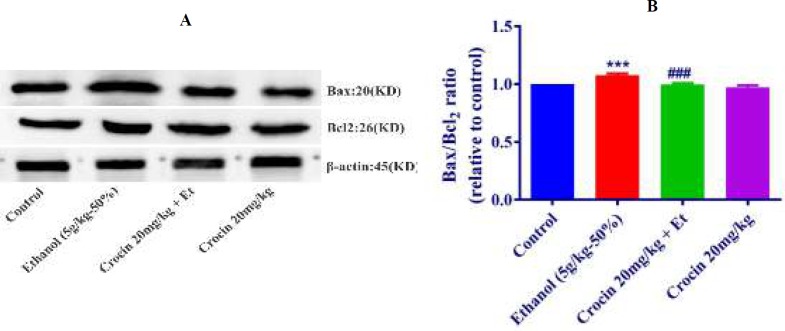
Effect of crocin 20 mg/kg and ethanol (Et) on the protein levels of Bax and Bcl-2 in the rat liver tissue. (A) Representative Western blots showing specific bands for Bax, Bcl-2 and β-actin as an internal control. Equal amounts of protein sample (50 μg) obtained from whole liver homogenate were applied in each lane. (B) Densitometric data of protein analysis. Data are expressed as the mean±SEM. *** *P<*0.001 comparison with control group, ### *P<*0.001 comparison with ethanol group, Tukey-Kramer test, n=4

**Figure 9:**
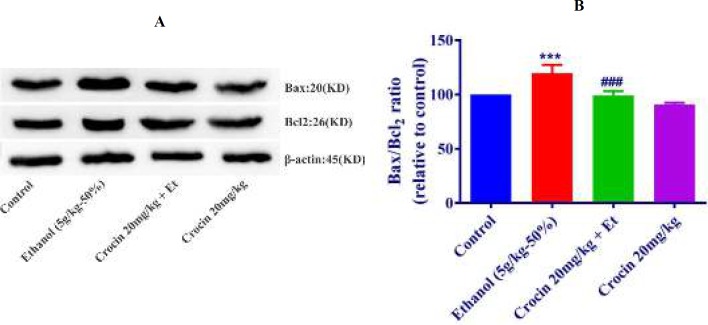
Effect of crocin 20 mg/kg and ethanol (Et) on the protein levels of Bax and Bcl-2 in the rat kidney tissue. (A) Representative Western blots showing specific bands for Bax, Bcl-2 and β-actin as an internal control. Equal amounts of protein sample (50 μg) obtained from whole kidney homogenate were applied in each lane. (B) Densitometric data of protein analysis. Data are expressed as the mean±SEM. *** *P<*0.001 comparison with control group, ### *P<*0.001 comparison with ethanol group, Tukey-Kramer test, n=4

**Figure 10:**
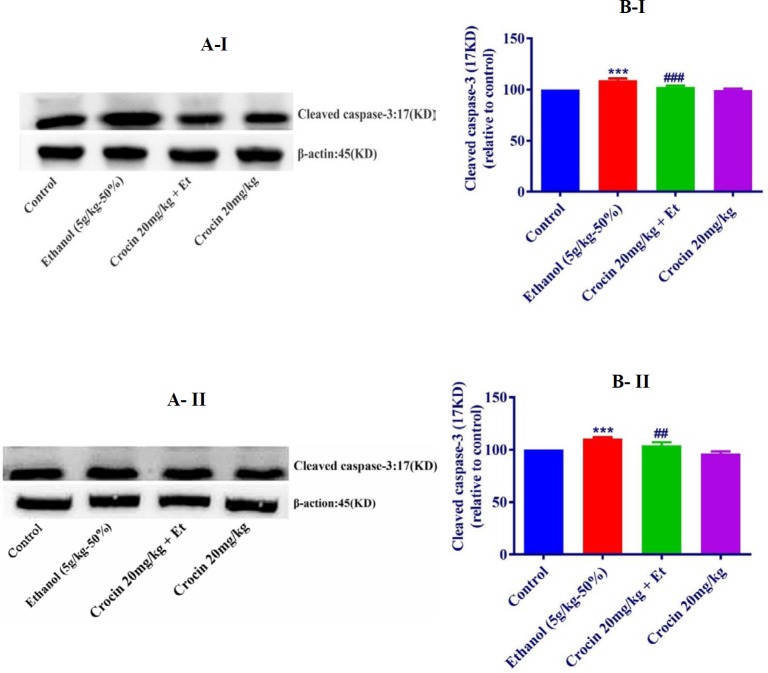
Effect of crocin 20 mg/kg and ethanol (Et) on the protein level of cleaved caspase-3 in the rat liver (I) and kidney (II) tissues. (A) Representative Western blots showing specific bands for cleaved caspase-3 (17KDa) and β-actin as an internal control. Equal amounts of protein sample (50 μg) obtained from whole liver and kidney homogenates were applied in each lane. (B) Densitometric data of protein analysis. Data are expressed as the mean±SEM. *** *P<*0.001 comparison with control group, ## *P<*0.01, ### *P<*0.001 comparison with ethanol group, Tukey-Kramer test, n=4

**Figure 11:**
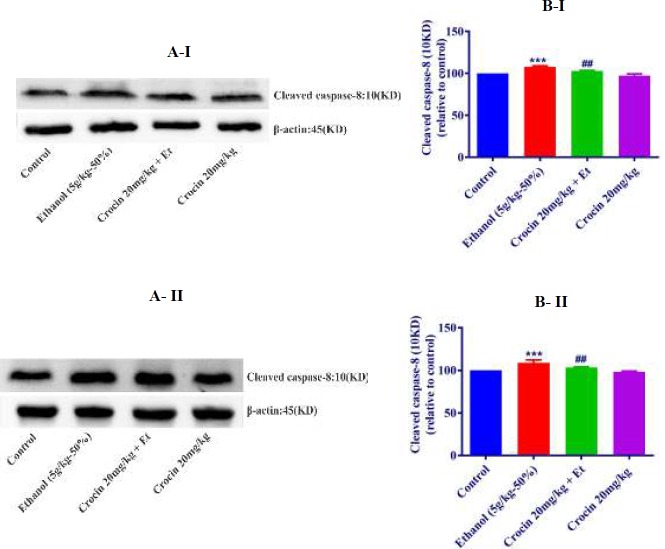
Effect of crocin 20 mg/kg and ethanol (Et) on the protein level of cleaved caspase-8 in the rat liver (I) and kidney (II) tissues. (A) Representative Western blots showing specific bands for cleaved caspase-8 (10KDa) and β-actin as an internal control. Equal amounts of protein sample (50 μg) obtained from whole liver and kidney homogenates were applied in each lane. (B) Densitometric data of protein analysis. Data are expressed as the mean ± SEM. *** *P<*0.001 comparison with control group, ## *P<*0.01 comparison with ethanol group, Tukey-Kramer test, n=4

**Figure 12:**
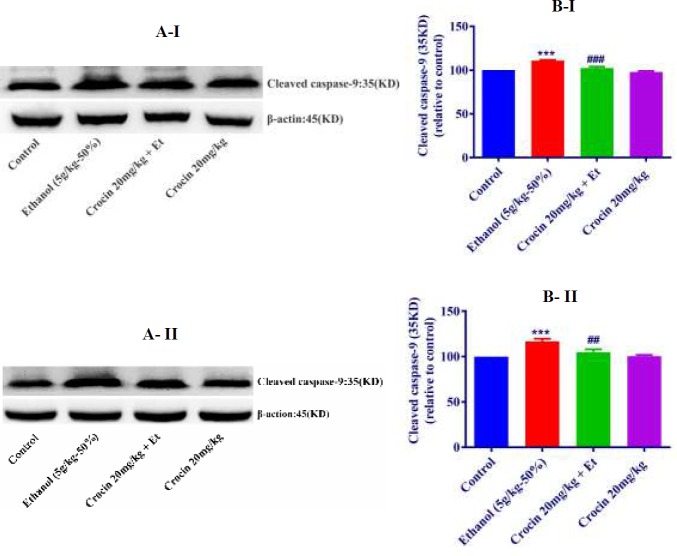
Effect of crocin 20 mg/kg and ethanol (Et) on the protein level of cleaved caspase-9 in the rat liver (I) and kidney (II) tissues. (A) Representative Western blots showing specific bands for cleaved caspase-9 (35KDa) and β-actin as an internal control. Equal amounts of protein sample (50 μg) obtained from whole liver and kidney homogenates were applied in each lane. (B) Densitometric data of protein analysis. Data are expressed as the mean±SEM. *** *P<*0.001 comparison with control group, ## *P<*0.01, ### *P<*0.001 comparison with ethanol group, Tukey-Kramer test, n=4

The protein expression of Bax/Bcl-2 was up-regulated in ethanol group in the liver and kidney (*P<*0.001) (Figure 8, Figure 9). Also, protein expressions of caspase-3, -8, and -9 were up-regulated by ethanol in the liver (*P<*0.001) and kidney* (P<*0.001) tissues (Figure 10, Figure 11, Figure 12, respectively). On the contrary, in liver and kidney, the Bax/Bcl-2 ratio (*P<*0.001) (Figure 8, Figure 9) and the levels of caspase-3 (*P<*0.001 and* P<*0.01, respectively), caspase-8 (*P<*0.01) and caspase-9 (*P<*0.001and* P<*0.01, respectively) (Figure 10, Figure 11, Figure 12 respectively) have declined because of simultaneous usage of ethanol and crocin 20 mg/kg in the kidney and liver.

***Measurement of mRNA level of Bax/Bcl-2 ratio in the liver and kidney tissues by quantitative RT-PCR***

**Figure 13:**
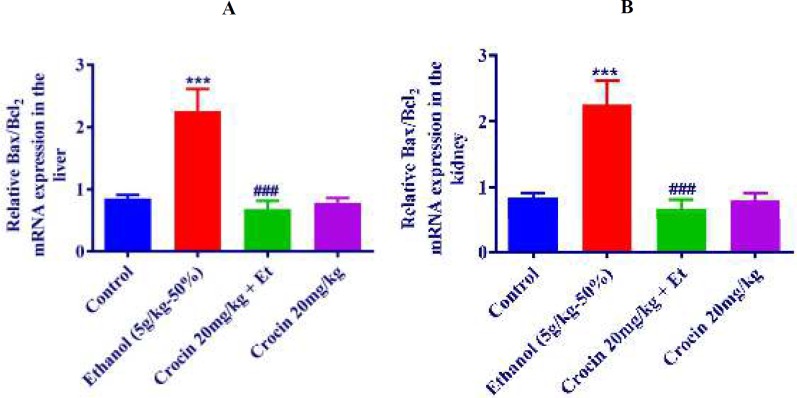
Effect of ethanol (Et) and crocin on Bax/Bcl-2 mRNA expression in the rat liver (A) and kidney (B) tissues after 4 weeks treatment by real-time PCR. The transcript level of each sample was normalized against β-actin transcript level. Data are expressed as the mean±SEM. *** *P<*0.001 comparison with control group, ### *P<*0.001 comparison with ethanol group, Tukey-Kramer test, n=4

The data proved that in both of liver and kidney, ethanol up-regulated Bax/Bcl-2 mRNA expression (*P<*0.001), while in crocin 20 mg/kg plus ethanol group, the expression of Bax/Bcl-2 mRNA was reduced and it was similar to the control group (Figure 13).

**Table 1:** Primary and secondary antibodies used for Western blot analysis of liver and kidney tissue

**Primary antibodies**	Rabbit monoclonal anti-serum against caspase-3 (Cell Signaling, #9665) Rabbit monoclonal anti-serum against caspase-8 (Cell Signaling, #4790) Rabbit monoclonal anti-serum against caspase-9 (Cell Signaling, #9506) Rabbit polyclonal anti-serum against Bax (Cell Signaling, #2772) Rabbit monoclonal anti-serum against Bcl_2_ (Cell Signaling, #2870) Mouse monoclonal anti-serum against β-actin (Cell Signaling, #3700)
**Secondary antibodies**	Anti-mouse IgG labeled with horseradish peroxidase (Cell Signaling, #7076) Anti-rabbit IgG labeled with horseradish peroxidase (Cell Signaling, #7074)

**Table 2:** Sequences of different primers used for real-time PCR reactions

**Gene**	**Primer**	**Sequence**
**Bcl**_2_	Forward	5’-GGTGGAGGAACTCTTCAGGGA-3’
	Reversed	5’-GGTTCAGGTACTCAGTCATCCA-3’
**Bax**	Forward	5’-TGCTGATGGCAACTTCAACT-3’
	Reversed	5’-ATGATGGTTCTGATCAGCTCG-3’
**β-actin**	Forward	5’-GGGAAATCGTGCGTGACATT-3’
	Reversed	5’-GCGGCAGTGGCCATCTC-3’

**Table 3:** Effects of ethanol (Et) and crocin (4 weeks) on biochemical marker levels such as ALT, AST, ALP, Cholesterol, TG, LDL, HDL, FBS, Urea and Creatinine in rats. Data showed as mean±SEM, * *P<*0.05, *** *P<*0.001 comparison with control, # *P<*0.05, ## *P<*0.01, ### *P<*0.001 comparison with ethanol treated group, Tukey-Kramer test, n=6

**Group**	**Control**	**Ethanol (5** **g/kg – 50% v/v)**	**Crocin** **10 mg/kg + Et**	**Crocin** **20** **mg/kg + Et**	**Crocin** **20** **mg/kg**	**Crocin** **40 mg/kg + Et**	**Crocin** **40 mg/kg**
**ALP** **(U/L)**	342.0 ± 7.703	443.4 ± 22.02	441.4 ± 51.56	337.0 ± 16.99	334.2 ± 27.79	334.2 ± 31.79	396.0 ± 15.70
**ALT** **(U/L)**	60.17 ± 1.833	75.40 ± 0.2449 *	79.00 ± 5.329	55.40 ± 2.358 **###**	47.80 ± 1.985	74.20 ± 3.929	50.80 ± 2.267
**AST** **(U/L)**	166.0 ± 1.506	224.6 ± 4.045 *	256.6 ± 15.44	163.2 ± 4.329 **#**	130.0 ± 2.000	233.4 ± 31.94	169.4 ± 7.318
**CHOL** **(mg/dL)**	67.50 ± 1.500	80.80 ± 5.229	73.40 ± 4.032	72.40 ± 4.654	51.40 ± 3.641	81.00 ± 5.030	69.40 ± 2.694
**TG** **(mg/dL)**	44.67 ± 4.702	85.60 ± 6.290 ***	60.80 ± 2.990 **##**	54.60 ± 3.572 **###**	50.40 ± 1.288	65.40 ± 5.963	55.40 ± 3.530
**HDL** **(mg/dL)**	27.17 ± 0.872	25.60 ± 1.122	27.80 ± 1.281	28.40 ± 1.077	28.00 ± 0.316	24.40 ± 1.600	24.40 ± 0.8124
**LDL** **(mg/dL)**	7.833 ± 0.307	9.600 ± 0.2449 *	8.000 ± 0.3162 **#**	6.800 ± 0.3742 **###**	6.600 ± 0.400	8.600 ± 0.4000	6.200 ± 0.3742
**FBS** **(mg/dL)**	89.83 ± 3.321	105.0 ± 2.582	108.6 ± 7.960	91.00 ± 5.992	88.60 ± 4.057	104.4 ± 6.524	88.80 ± 1.772
	32.41 ± 2.532	51.38 ± 5.137 *	47.36 ± 2.759	36.06 ± 3.392 #	34.14 ± 2.247	46.92 ± 3.652	43.73 ± 1.851
**Creatinine (mg/dL)**	0.64 ± 0.361	1.08 ± 0.137 *	0.92 ± 0.726	0.71 ± 0.528 #	0.67 ± 0.692	0.87 ± 0.146	0.81 ± 0.239

**Table 4:** Effect of crocin and ethanol (Et) on histopathological changes in liver and kidney tissues of rat after 4 weeks treatment. Histopathological criteria were determined semi-quantitatively from mild (+) to moderate (++) and sever (+++), n=6

**Groups**	**Liver**	**Kidney**
**Control**	Normal	Normal
**Ethanol (5 g/kg – 50% v/v)**	Severe congestion (+++), steatosis (+++) and portal lymphocytic infiltration (++)	Proteinuria (++) and infiltration of inflammatory factors around glomerular (++)
**Crocin 10 mg/kg + ethanol**	Moderate congestion (++), steatosis (+) and portal lymphocytic infiltration (+)	Proteinuria (+) and infiltration of inflammatory factors around glomerular (+)
**Crocin 20 mg/kg + ethanol**	Mild congestion (+)	Normal
**Crocin 20 mg/kg**	Normal	Normal
**Crocin 40 mg/kg + ethanol**	Severe congestion (+++), steatosis (+) and portal lymphocytic infiltration (++)	Proteinuria (++) and infiltration of inflammatory factors around glomerular (+)
**Crocin 40 mg/kg**	Mild congestion (+) and portal lymphocytic infiltration (+)	Proteinuria (+) and infiltration of inflammatory factors around glomerular (+)

## Discussion

The development of oxidative stress by ethanol is recognized to contribute to the beginning of serious irregularities in a variety of body parts namely liver and kidney (32, 33). Nowadays, herbal drugs are used for treatment of diseases induced by ethanol since they are useful, safe and effective. *C. sativus *L. (saffron) is one of the famous plants because of traditional knowledge of medicinal properties and various therapeutic effects according to the biological and pharmacological potential of saffron. A wide range of modern pharmacological studies as well as clinical experiments confirms that saffron is an interesting agent against oxidative stress, lipid peroxidation and apoptosis (12, 15). Due to all the aforementioned positive properties, in the present investigation, crocin (major bioactive ingredient of saffron) was used to reduce ethanol toxicity. According to the results of our research, ethanol led to renal toxicity and hepatotoxicity via increasing the levels of biochemical markers (urea, creatinine, LDL, TG, AST, and ALT levels) and inflammatory biomarkers (IL-6 and TNF-α), GSH content decline and the rise of MDA levels, extensive disturbance in gene expression and apoptosis while we proved that crocin treatment was able to remove all above mentioned abnormalities in liver and kidney of rat in a period of 28 days.

GSH is regarded as a major antioxidant in organisms with the capacity to minimize ROS-induced damage and MDA is considered as a symptom of oxidative stress. GSH decline and MDA overproduction in ethanol consumption are induced by appearance of oxidative stress in addition to increase in free radicals (34). Our study revealed that ethanol dependence significantly reduced the content of GSH and increased the MDA level in the kidney and liver. In the same vein, Amrani *et al.* demonstrated that GSH levels were diminished and MDA was heightened in liver and heart through ethanol consumption (5 g/kg, 40% v/v) every 12 hr (35). Additionally, another study substantiated our findings by revealing that a 30-day ethanol administration (40% w/v, 2 g/kg/day) diminished GSH levels and heightened MDA in the kidney (36). Likewise, in a 30-week experiment in male Wistar rats, one ml of ethanol (35% w/w, 2 g/kg BW) was used. Based on the findings, the levels of MDA and GSH in rat kidneys illustrated abnormal fluctuations (37). Moreover, a 15-day administration of 12 ml of ethanol (50%) demonstrated identical impacts on MDA and GSH in liver; however, 4 ml of watermelon juice ameliorated the negative effects of ethanol on liver (38). Similar to ethanol effects, some agents (methotrexate and cisplatin) cause significant abnormalities in the levels of MDA and GSH, because they can induce oxidative stress and toxicity in different tissues (39-41). In this connection, for assessment of myricetin and casticin therapeutic properties on liver damage induced by methotrexate, several parameters were evaluated such as MDA levels, activities of antioxidant enzymes SOD, GPX, CAT and expression of caspase-3 and 8-OHdG. The analysis of data proved that the level of MDA and expression of caspase-3 and 8-OHdG were increased and reduction of SOD, CAT, and GPX activities were observed because of oxidative stress induced by methotrexate. While, casticin and myricetin could moderate them by DNA protective, increasing of activity/production of antioxidant enzymes, alleviation of injury caused by ROS production and anti-apoptotic properties (42). In our study crocin (20 mg/kg, 28 days) restored the toxic effects of ethanol on MDA and GSH contents. 

Similar to crocin, *Penthorum chinense* Pursh (43), *Nigella sativa *(32), *Tetracarpidium conophorum* (44) and ginger extract (45) have been acknowledged to possess defensive properties against unusual features in MDA and GSH levels caused by ethanol. The histopathological findings showed that ethanol might lead to proteinuria and penetration of inflammatory factors around glomerular in the kidney in addition to portal lymphocytic infiltration, moderate steatosis, and critical congestion in liver. The findings of the present study substantiate the reports demonstrating that 28-day administration of ethanol (3 g/kg/day) resulted in critical congestion, moderate hematuria, and infiltration of inflammatory focal adjacent glomerular in the kidney; as well as moderate infiltration of inflammatory focal portal space and steatosis and critical congestion in the liver (32, 33). According to results of our study, crocin as a protective agent alleviated pathological damages in the alcoholic rat. This result was in agreement with another study investigating the effects of *N. sativa *fixed oil and thymoquinone on toxicity of ethanol in liver and kidney (32, 33). In our research, subsequent to a one-month administration of ethanol (50% v/v - 5 g/kg), levels of creatinine and urea (kidney damage markers), LDL, TG, AST, and ALT levels were escalated. Dose and exposure time of ethanol is influential in fluctuation of these factors. For instance, biochemical factors undergo more detrimental effects in administration of ethanol (20%, 5 g/kg BW) for 60 days (46), than consumption of ethanol (30%, 3 g/kg BW) for 15 days (47). In addition, a wealth of research indicated that AST, ALT, and ALP are ameliorated via toxicity of ethanol. For instance, consumption of ethanol (7.9 g/kg/day) for 45 days leads to an oxidative damage which heightens the GGT, AST, ALT, and ALP activates (48). Another study declared that a nine-week administration of ethanol (56% v/v, 10 ml/kg) induced decline in HDL-C level as lipid disorders, enhanced LDL-C, TG levels and cholesterol, increased AST and ALT activities as unusual liver performance (49). To evaluate renal function, we measured the plasma urea and creatinine that increasing of these parameters confirms the initial kidney dysfunction due to ethanol consumption. According to analysis of data, ethanol toxicity could cause fluctuation in these parameters. The levels of urea and creatinine demonstrated an increasing trend in ethanol group, while crocin treatment reduced them considerably. These results proved that crocin is able to neutralize toxic effects of ethanol in the kidneys. Similar to our study, the use of ethanol (20%, 5 g/kg/day) elevated the concentration of urea and creatinine in rat plasma during 60 days. But treatment of *Pterocarpus santalinus* (250 mg/ kg/day) lowered these items (50). In another research, urea and creatinine were considered as sensitive markers of toxicity in the kidney. The administration of ethanol (30%, 3 g/kg/day) for a period of 15 days caused the increasing trend in the levels of creatinine and urea in rat while they were moderated in protective group by sardinelle (*Sardinella aurita*) protein hydrolysate (47). Also, the creatinine was assessed as a nephrotoxic index in chronic ethanol treatment (30% v/v, 8 weeks). The fluctuation of creatinine in alcoholic rats was introduced as an oxidative effect of chronic ethanol consumption on the kidney functions that folic acid supplementation improved it (5). The results obtained from our experience demonstrated that levels of cholesterol, FBS, HDL, and ALP did not show meaningful differences between control and ethanol group. These parameters might be heightened if the time and amount of ethanol administration is increased. With regards to the findings of the study, the release of cytokines namely IL-6 and TNF-α might be induced via oxidative stress which is regarded as a significant markers of tissue inflammation (51). The current research authenticated a substantial increase in the TNF-α and IL-6 levels in liver in the group with ethanol administration in comparison with the control group; however, no substantial difference in groups was detected in the kidney. It is believed that the levels of IL-6 and TNF-α in the kidney might be affected by an increase in amount and time of ethanol administration. The findings of this study are consistent with other bodies of research illustrating IL-6 and TNF-α as proper signs of inflammation in fatty liver and hepatic lipid accumulation, substantially heightened as a result of ethanol administration (5 g/kg - 12 weeks or 2 g/kg for 30 days) in rats (36). Similarly, another survey, conducting on AML12 cells, substantiated that ethanol administration increased TNF-α expression, NOX4 (oxidative stress signs) expression, and GSH-synthesizing enzymes expression. Besides other detrimental impacts of ethanol, it has been revealed that an increase in TNF-α level in cell line of liver is as a result of inflammation (52). Conversely, *Amorphophallus campanulatus*, *P. chinense* Pursh, and *Akebia quinata* extracts decreased the elevation of IL-6 and TNF-α levels (36, 43, 52). 

The effect of crocin (20 mg/kg) was consistent with aforementioned plants that can alter the affected TNF-α and IL-6 levels by ethanol. Moreover, previous research confirmed that* Antrodia camphorata* and *Armillariella tabescens* (0.5 g/kg), *P. chinense* Pursh (10.30 g/kg) and green *Capsicum annum* (250 mg/kg) have shown a protective mechanism against ethanol toxicity (5 g/kg, a modified Lieber-Decarli alcohol liquid diet, 2 g/kg respectively) similar to silymarin (positive control) in amounts of 200, 86 and 50 mg/kg respectively (53, 43, 54). Therefore, it can be concluded that like silymarin, crocin reveals the protective effect against liver toxicity induced by ethanol (50% v/v - 5 g/kg).

In our experiment, we evaluated the levels of caspases-3, -8, -9 and Bax/Bcl-2 ratio in order to assess apoptosis. Treatment with crocin (20 mg/kg/day) diminished the increased level of Bax/Bcl-2 ratio in the mRNA and protein levels in the liver and kidney. Also, the increasing of caspase-8, -9, and -3 was prevented by crocin. Consistent to our findings, β-carotene (1 μM) stopped the hepatotoxicity stimulated by ethanol through anti-apoptotic and antioxidant aspects. β-carotene ameliorated the levels of caspase-9, caspase-3, expressions of CYP2E1 and lipid peroxides (55). In this regard, Liu *et al.* confirmed that ethanol (300 mmol/l) could increase Bax level, decline in the level of Bcl_2_, and disturb the activation of caspase-3 which was consistent with our findings (56). Also, thymoquinone (10 mg/Kg/day) and *N. sativa *fixed oil (0.5 ml/kg/day) were revealed to improve the mRNA and protein levels of Bax/Bcl-2 ratio and the levels of caspase-8, caspase-9, caspase-3 which stimulated by ethanol (40% v/v) at the dose of 3 g/kg/day for 4-week in liver and kidney of rats (32, 33). According to the recent study, treatment with saffron aqueous extract (80 mg/kg) for 4 weeks was useful against the abnormal changes of the mRNA and protein levels of Bax/Bcl-2 ratio and the levels of caspase-3, caspase-8 and caspase-9 induced by ethanol (50% v/v-5 g/kg) in the liver of rat (57). In general, it can be said that although ethanol increased Bax/Bcl-2 ratio and the levels of caspase-3, -8, -9, crocin as the main bioactive component of saffron could stop induction of apoptosis by ethanol.

## Conclusion

According to all obtained data, crocin has protective effects against nephrotoxicity and hepatotoxicity stimulated by ethanol through prevention of apoptosis by adjusting the levels of caspase-3, -8 and -9, ameliorating Bax/Bcl-2 ratio (protein and mRNA), alleviating biochemical levels (LDL, TG, AST, ALT, creatinine and urea) and inflammatory biomarkers (IL-6 and TNF-α), reducing histopathological damages, diminishing lipid peroxidation, and increasing GSH content.
